# Applying User-Centered Design and Implementation Science to the Early-Stage Development of a Telehealth-Enhanced Hybrid Cardiac Rehabilitation Program: Quality Improvement Study

**DOI:** 10.2196/47264

**Published:** 2023-07-13

**Authors:** Andrea T Duran, Adrianna Keener-DeNoia, Kimberly Stavrolakes, Adina Fraser, Luis V Blanco, Emily Fleisch, Nicole Pieszchata, Diane Cannone, Charles Keys McKay, Emma Whittman, Donald Edmondson, Rachel C Shelton, Nathalie Moise

**Affiliations:** 1 Center for Behavioral Cardiovascular Health Columbia University Irving Medical Center New York, NY United States; 2 New York Presbyterian Hospital New York, NY United States; 3 Mailman School of Public Health Columbia University New York, NY United States

**Keywords:** user-centered design, implementation science, cardiac rehabilitation, telemedicine, remote patient monitoring, behavioral intervention development, hybrid

## Abstract

**Background:**

Cardiac rehabilitation (CR) is an evidence-based intervention that improves event-free survival in patients with cardiac conditions, yet <27% of all eligible patients use CR in the United States. CR is traditionally delivered in clinic-based settings where implementation barriers abound. Innovative nontraditional program designs and strategies are needed to support widespread CR uptake.

**Objective:**

This study aimed to demonstrate how user-centered design (UCD) and implementation science (IS) principles and methods can be integrated into the early-stage development of nontraditional CR interventions.

**Methods:**

As part of a NewYork-Presbyterian Hospital (NYPH) quality improvement initiative (March 2020-February 2022), we combined UCD and IS principles and methods to design a novel home- and clinic-based telehealth-enhanced hybrid CR (THCR) program. We co-designed this program with multilevel stakeholders using an iterative 3-step UCD process to identify user and contextual barriers and facilitators to CR uptake (using semistructured interviews and contextual inquiry [step 1]), design an intervention prototype that targets contextual and user factors and emulates the evidence-based practice (through design workshops and journey mapping [step 2]), and review and refine the prototype (according to real-world usability testing and feedback [step 3]). The UCD process was informed by the Theoretical Domains Framework and Consolidated Framework for Implementation Research.

**Results:**

At step 1, we conducted semistructured interviews with 9 provider- and system-level stakeholders (female: n=6, 67%) at 3 geographically diverse academic medical centers, which revealed behavioral (eg, self-efficacy and knowledge) and contextual (eg, social distancing guidelines, physical space, staffing, and reimbursement) barriers to uptake; hybrid delivery was a key facilitator. Step 2 involved conducting 20 design workshops and 3 journey-mapping sessions with multidisciplinary NYPH stakeholders (eg, digital health team, CR clinicians, and creative director) where we identified key design elements (eg, mix of clinic- and home-based CR and synchronous remote patient monitoring), yielding an initial THCR prototype that leveraged NYPH’s telehealth infrastructure. At step 3, we conducted usability testing with 2 CR clinicians (both female) administering home-based sessions to 3 CR patients (female: n=1, 33%), which revealed usability themes (eg, ease of using remote patient monitoring devices or a telehealth platform, technology disruptions, and confidence in using the telehealth platform to safely monitor patients) and design solutions (eg, onboarding sessions, safety surveys, and fully supervised remote sessions) to be included in the final THCR prototype.

**Conclusions:**

Combining UCD and IS methods while engaging multidisciplinary stakeholders in an iterative process yielded a theory-informed THCR program targeting user and contextual barriers to real-world CR implementation. We provide a detailed summary of the process and guidance for incorporating UCD and IS principles and methods into the early-stage development of a nontraditional CR intervention. The feasibility, acceptability, appropriateness, and usability of the final THCR prototype is being evaluated in an ongoing study.

## Introduction

### Background

Cardiac rehabilitation (CR), which involves exercise training, patient education, and health behavior modification in clinic- and home-based settings, is a class I intervention, with a level A recommendation for secondary prevention among patients with cardiac conditions [1]. Despite the well-established effectiveness of CR [2-4], <27% of eligible patients with cardiac conditions participate in, and adhere to, CR programs in the United States. Undoubtedly, barriers to implementing *traditional* (ie, clinic based) CR have been identified (eg, transportation, time, scheduling, and motivation) [5-7], with new implementation barriers emerging during the COVID-19 pandemic (eg, social distancing and patient fear) [8-12]. Because of the sustained low rates of participation in clinic-based CR programs, a 2019 scientific statement from expert organizations highlighted an urgent need to identify *nontraditional* models (eg, home based, alternative site based, and hybrid) to improve CR participation and enhance widespread reach [13].

Over the past 2 decades, nontraditional CR models have emerged as viable solutions owing to their ability to overcome common patient-level barriers to clinic-based CR (eg, transportation and time) and improve clinical and health-related quality-of-life outcomes with effect sizes similar to those of clinic-based CR [13-15]. However, great variability in program design and barriers to uptake exist (distinct from barriers to clinic-based CR), including patient safety concerns, effective patient-provider communication, and inconsistent reimbursement of remote and home-based sessions [13]. To overcome these barriers and ensure continuity of CR in the COVID-19 era, national and international scientific statements called for broader use of information and communication technologies (ie, telehealth [eg, websites and mobile phone apps]) to deliver and integrate nontraditional CR into health care settings [8,16].

Although widespread support for telehealth-enabled nontraditional CR exists, determining which design elements to include for a specific organizational and health care infrastructure and patient population remains unclear, partly because the multilevel factors that impede or support the successful implementation of such programs are not well defined. Furthermore, the optimal design of a nontraditional CR program (eg, the frequency of home-based sessions and type of user-friendly telehealth devices) to improve patient and provider experiences as well as clinical outcomes in racial and ethnic and socioeconomically diverse settings has yet to be established [17]. These myriad sources of uncertainty illustrate the need for guidance on how best to design nontraditional CR programs that address barriers and facilitators to uptake not only at the patient level but also at the provider and health care system levels. The challenges outlined make this gap (between the evidence of CR effectiveness and the uptake of CR programs in real-world settings) particularly ripe for an intervention development approach that combines user-centered design (UCD) principles, which use an iterative and highly stakeholder-engaged process to cocreate products that are directly responsive to the end-user experience [18,19], and implementation science (IS), which uses theoretical frameworks to target implementation barriers and elucidate key mediators or moderators of implementation outcomes (eg, feasibility and acceptability) [20,21], particularly at early stages of intervention development and refinement [22,23].

### Objectives

Despite prior calls for combining UCD and IS [24-26], few applied research examples (and fewer for CR) demonstrating how to approach and operationalize this process exist. Using a NewYork-Presbyterian Hospital (NYPH) quality improvement (QI) project focused on improving CR uptake as a use case, we describe how we infused UCD principles and IS methods to develop a telehealth-enhanced hybrid CR (THCR) program at stage I of the National Institutes of Health (NIH) Stage Model for Behavioral Intervention Development [22,27]. To our knowledge, this is one of the first studies to describe a theory-informed iterative approach to the design and implementation of a THCR program in a real-world academic medical setting, particularly at NIH stage I intervention generation, refinement, and pilot-testing. Our overarching goal is to improve the routine and equitable uptake of nontraditional CR programs at the patient, provider, and system levels.

## Methods

### Evidence-Based Practice and Context

Traditional CR is an evidence-based, standard-of-care, and clinic-based program that includes patient assessment (medical history and functional capacity), exercise training (aerobic and strength), patient education and counseling (nutrition and psychosocial), and risk factor management (lipids, blood pressure [BP], weight, diabetes mellitus, and smoking) [28]. The traditional CR model offers up to 36 sessions over 3 to 6 months at a facility (eg, hospital or clinic). Each CR session is 31 to 60 minutes in duration, often occurs in group-based settings (eg, 2-4 people), and is directly supervised in person by a team of CR clinicians (eg, physical therapist [PT], nurse, and exercise physiologist). Nontraditional CR targets the same core components as traditional CR but delivers CR sessions outside of the traditional clinic-based setting. Nontraditional CR programs can include home-based, virtual, telehealth, telemedicine-based, and community-based or unsupervised CR sessions or a combination of such sessions with at least 1 center-, facility-, or clinic-based or supervised CR session (ie, *hybrid* CR) [13,29,30]. Although nontraditional models have been encouraged, traditional clinic-based CR remains the most widely available across the United States and is established as a reimbursable service by the Centers for Medicare & Medicaid Services [31,32]. The COVID-19 pandemic halted many clinic-based CR services in the United States and created opportunities for the rapid adoption of reimbursable telemedicine-enhanced programs [10].

### Study Overview

#### QI Project

**Figure 1:**
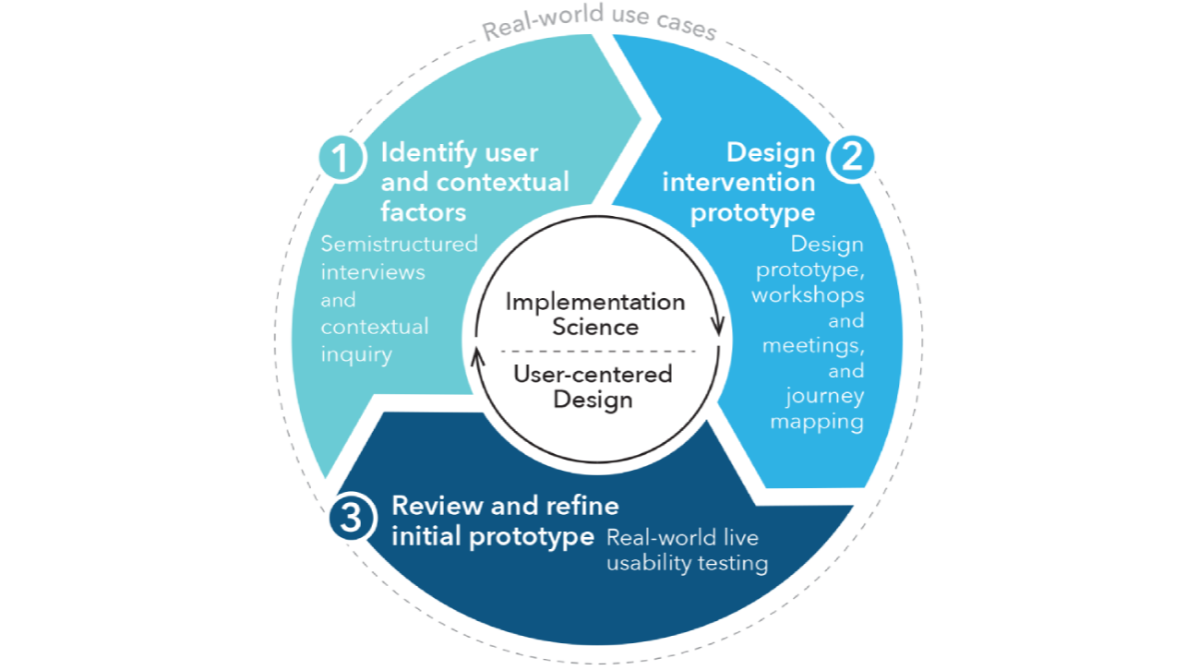
Overview of combined user-centered design and implementation science process to develop a nontraditional cardiac rehabilitation program.

As part of an NYPH QI project (March 2020 to February 2022), we (ie, faculty and staff affiliated with Columbia University Irving Medical Center) worked alongside the NYPH’s department of rehabilitation medicine to design a nontraditional CR program to offer patients in the post–COVID-19 era. First, we established a design concept, which was to develop a nontraditional CR program that can (1) emulate the same core components of the evidence-based practice to retain similar program effectiveness, (2) reduce user and structural barriers to improve multilevel implementation, and (3) improve experiences of both patients and clinicians to optimize the usability of the nontraditional CR prototype compared with the traditional CR model. We then engaged patient-, provider-, and system-level stakeholders in an iterative three-step UCD process to (1) identify user and contextual factors that could influence uptake (using semistructured interviews and contextual inquiry), (2) design an intervention prototype (through design team workshops and journey mapping), and (3) review and refine the intervention prototype (according to real-world user testing and feedback). The 3-step UCD process was modeled after the conceptual model presented by Haines et al [33]. To guide the UCD process, we used the Theoretical Domains Framework (TDF; 84 theoretical constructs within 14 domains [eg, knowledge, skills, and social or professional role]) [34-36] and the Consolidated Framework for Implementation Research (CFIR; 39 constructs within 5 domains [eg, inner setting and outer setting]) [37,38]. Although there is considerable overlap in these theoretical IS frameworks, we leveraged the ways in which the TDF addresses individual-level determinants (eg, motivation and capability), and the CFIR addresses system-level determinants (eg, inner or organizational setting and outer or policy setting) [39]. We applied the revised Standards for Quality Improvement Reporting Excellence (SQUIRE 2.0) guidelines when preparing this manuscript [40]. Figure 1 provides a conceptual model of the combined UCD and IS process and methods. Table S1 in Multimedia Appendix 1 [35,38,41-50] provides descriptions and definitions of key UCD and IS methods, frameworks, and terms included throughout the design process (eg, CFIR, TDF, journey mapping, usability testing, and contextual inquiry).

#### Step 1 Methods: Identify User and Contextual Factors

From March 2020 to July 2020, we conducted semistructured interviews with key stakeholders with expertise in CR as well as in-depth interviews with providers from NYPH as a form of contextual inquiry [41]. The goal of this step was to understand barriers and facilitators to CR implementation that emerged during the COVID-19 pandemic and acquire information necessary to optimize the adoption of CR in the era of remote clinical care.

##### Theory-Informed Semistructured Interviews

We conducted semistructured key informant interviews via video, telephone, or in person with researchers, clinicians, and administrators with expertise in CR (clinic or home based). These key stakeholders (ie, researchers, clinicians, and administrators) were identified as interviewees using a combination of academic literature review (eg, scientific statements and home-based CR programs) and snowball sampling. Each interview was conducted in a rapid-cycle iterative process using open-ended questions that explored barriers and facilitators to CR implementation during the pandemic. Interview notes were coded into themes for clinic- and home-based CR by the lead author (ATD) using inductive thematic analysis [51]. Informed by the TDF and CFIR domains and constructs, the lead author (ATD) and senior author (NM) then categorized each theme into behavioral (capability and motivation) and contextual (inner setting and outer setting) determinants, respectively, of clinic- and home-based CR implementation. Both the lead author and senior author have training in IS (theories, models, and frameworks) and qualitative methods.

##### Contextual Inquiry

In parallel to conducting semistructured interviews, we completed in-depth interviews with key clinician stakeholders at NYPH [52,53]. We used purposive sampling to identify members with direct experience of administering CR and similar programs that use telemedicine at NYPH. Each in-depth interview aimed to understand the CR workflow, user and contextual factors to CR implementation, patient population (eg, sociodemographics and digital health literacy), and the use of telemedicine within the context of NYPH.

#### Step 2 Methods: Design Initial Intervention Prototype Based on User and Contextual Factors

From May 2020 to March 2021, we engaged a design team in an iterative series of design team prototyping workshops or meetings [42] and journey mapping sessions [43,44] to develop an initial nontraditional CR program prototype that preserved the effectiveness of traditional CR and fit the context of our racial and ethnic and socioeconomically diverse setting while optimizing feasibility, appropriateness, and usefulness [54].

##### Journey Mapping and Design Team Prototyping Workshops or Meetings

First, we assembled a 6-member NYPH design team of CR clinicians (n=3, 50%), digital health team members (n=2, 33%), and a creative director with expertise in IS and UCD (n=1, 17%). Next, we engaged the design team in an iterative process of journey mapping and prototyping workshops to develop a nontraditional CR prototype that addressed the barriers and leveraged the facilitators from step 1. The process began with visualizing key prototype design features (eg, home-based telemonitoring and exercise equipment, educational videos, and frequency of CR sessions), followed by journey mapping of the patient experience (eg, receiving the nontraditional CR program) and the CR clinician experience (eg, administering the program) from the beginning to end of program participation. Journey maps were presented to the design team members to stimulate engagement, deeper understanding of the challenges and opportunities, and discussion on how best to approach subsequent activities and design steps, while keeping the end users (ie, patient and clinician) at the center of the design. In the case where follow-up from workshop sessions was needed and to accommodate busy schedules, documented email threads were used to continue design feedback and prototype creation among various design team stakeholders.

##### Initial Intervention Prototype

We continued the iterative process of journey mapping and design team sessions until we obtained an initial prototype that incorporated core program components of the evidence-based practice (eg, exercise and education), key program design elements (eg, number and types of sessions), patient- and provider-facing protocols (eg, timing and details of sessions), and a telehealth monitoring protocol and platform (eg, how to navigate the telehealth platform).

#### Step 3 Methods: Review and Refine Initial Intervention Prototype

From April 2021 to January 2022, we conducted a series of usability testing sessions with real-world CR clinicians and CR patients, followed by a final round of design team workshops and journey mapping to refine the intervention prototype [45,46]. The goal of this step was to optimize usability and acceptability.

##### Usability Testing

Real-world usability testing of the initial prototype’s home-based CR protocol and telehealth monitoring platform was conducted at an NYPH CR clinic located in the Washington Heights neighborhood of New York City [46]. CR clinicians were included if they (1) were a full-time equivalent CR staff member and (2) provided CR treatment to patients attending the NYPH CR clinic. CR patients were included if they (1) were enrolled in CR at the NYPH CR clinic as part of their standard-of-care secondary prevention treatment and (2) expressed interest in user testing the nontraditional CR prototype. CR clinicians user tested navigating the telehealth platform (eg, identification of the patient calendar, surveys, measurements, and video call feature) to remotely administer (2-way video call) and monitor (heart rate [HR] and BP measurements) patients during the nontraditional CR sessions. CR patients user tested interacting with the telehealth devices (eg, tablet device, pulse oximeter, and BP monitor and cuff) to communicate with or view the CR clinician (2-way video call) and take vital sign measurements (HR and BP) before, during, and after aerobic and resistance exercise. Direct observations and field notes were used to document the patient-provider interaction and experiences with the equipment (eg, telehealth devices and exercise equipment), telehealth platform, and protocol design or flow.

##### Archival Analysis, Design Workshops, and Journey Mapping

After each usability session, the design team met to review what worked and did not work and problem-solved accordingly. Between the design team workshops, archival analysis (eg, characterize text from archived documents) of observation field notes, meeting minutes or notes, and emails were used to inform prototype and journey map revisions necessary to improve clinician- and patient-facing experiences [47,55]. The archived documents were analyzed by the lead author (ATD) and second author (AK-D) using thematic analysis and coded for factors that influenced patient and provider experiences related to equipment and the protocol (ie, navigation, visibility, and workflow). To ensure that our themes aligned with UCD principles, we mapped each theme to key usability constructs (eg, learnability, efficiency, memorability, error reduction, satisfaction, and exploitation of natural constraints) [26,56]. The usability themes guided the design team workshops and protocol refinement process, leading to ideal journey maps for each user experience (Figure S1 in Multimedia Appendix 1).

### Ethics Approval and Data Collection and Management

Ethics approval to use the data for research purposes was obtained from the Columbia University Irving Medical Center institutional review board (IRB-AAAT2306) and included a waiver of informed consent and Health Insurance Portability and Accountability Act authorization. All data were collected as part of the NYPH QI project and maintained using clinical QI and Health Insurance Portability and Accountability Act–compliant methods. Data were anonymized, and all personal identifiers were removed.

## Results

### Overview

From March 2020 to January 2022, we completed an iterative, multilevel-stakeholder, IS-informed, and 3-step UCD cycle that yielded a THCR prototype. The final nontraditional CR prototype embodied the following design concepts: (1) target user and contextual barriers and facilitators to CR implementation, (2) emulate the same core components of the evidence-based practice, and (3) refine the design in response to usability themes that emerged from clinician and patient experiences with the home-based CR protocol and telehealth platform. Details of the stakeholders involved in the design process are provided in Table S2 in Multimedia Appendix 1. In the following subsections, we provide an overview of the results from each step that led to the final THCR prototype.

### Step 1 Results: User and Contextual Factors of CR Implementation

#### Clinic- and Home-Based CR Determinants

We contacted 10 key stakeholders at 3 geographically diverse academic medical centers (in New York, California, and Michigan) and 9 (90%) agreed to participate in semistructured interviews (female: n=6, 67%; CR supervisors or directors: n=2, 22% [PT: 1/2, 50%, and PhD researcher: 1/2, 50%], health system leaders: n=2, 22% [PT site director: 1/2, 50%, and department chair, doctor of medicine: 1/2, 50%]; and clinician or staff: n=5, 56% [PT: n=2, 40%; registered nurse: n=1, 20%; exercise physiologist: n=1, 20%; and patient navigator: n=1, 20%]). The determinants of CR implementation categorized by the CFIR and TDF domains are presented in Table 1.

For clinic-based CR, key contextual barriers (CFIR or TDF construct, ie, *determinant theme*[*s*]; Table 1) to implementation included external policies (*social distancing guidelines*; eg, “Total volume of clinic-based CR patients will decrease due to social distancing”), patient needs and resources (*overwhelmed health care system*; eg, “[CR clinicians were] redeployed to inpatient side”), structural characteristics or available resources (*limited space/staff*; eg, “We don’t have much physical space” and “Per diem nurse started and she can’t go in clinic”), compatibility (*one-on-one sessions*; eg, “We can’t see people in groups anymore”), and low relative priority (*nonessential service*; eg, “We temporarily closed on-site exercise and appointments”). Emotion (*patient fear*; eg, “Patients were nervous to come in”; and *provider burnout*; eg, “Small [staff] capacity and long wait list”) was a key behavioral barrier for clinic-based CR.

For home-based CR, key contextual barriers to implementation included external policies (*reimbursement*; eg, “As of now, this is a completely free service...because it cannot be reimbursed” and “[We are] delivering video visits for free”), available resources (*telehealth services/devices/exercise equipment*; eg, “[CR clinicians] aren’t offering any equipment, [they are] working with what the patients already have” and “The quality of video is highly dependent on the strength of [the patient’s] Wi-Fi signal”), and compatibility (*one-on-one sessions*; eg, “A lot of [electronic/telehealth] applications in the hospital are meant to be one-on-one”). Key behavioral barriers to home-based CR related to knowledge (*unfamiliarity with home-based* CR*/telemedicine*; eg, “[Patients] have low health and technology literacy”), beliefs about capabilities *(ability to administer home-based CR*; eg, “[Clinicians] need to have better clinical skills to monitor remote patients”), beliefs about consequences (*patient safety*; eg, “[People still ask] what is the safety of the service?”), and decision-making (*triaging patients*; eg, “How do you decipher which ones get it first?”).

A key facilitator for CR in general was the use of a hybrid delivery model because it addressed select barriers to clinic- and home-based CR. Key facilitators for home-based CR included collaborating with hospital administration, CR, telehealth champions, or opinion leaders (eg, “Get buy-in from the leadership”), leveraging existing CR workflow or electronic health record (EHR) and telemedicine infrastructure and initiatives (eg, “[A home-based CR program] is consistent with the [hospital] goals to expand telemedicine and aligns well with the [hospital] telehealth initiative”), and intervention adaptability (eg, “Create as you go”).

**Table 1:** Multilevel determinants of cardiac rehabilitation implementation during step 1 categorized by the Consolidated Framework of Implementation Research (CFIR) and Theoretical Domains Framework (TDF) domains.

Framework, domain, and construct				Cardiac rehabilitation determinant themes		
				Clinic based		Home based or telehealth
**CFIR**						
	**Outer setting**					
		External policies	Social distancing guidelines		Reimbursement	
		Patient needs and resources	Overwhelmed health care system		N/A^a^	
	**Inner setting**					
		Relative priority	Nonessential service and provider redeployment		N/A	
		Structural characteristics	Limited number of staff		N/A	
		Available resources	Limited physical space and limited staff capacity		Limited staff capacity and hospital budget; lack of telehealth services, devices, and Wi-Fi access; and lack of home-based exercise equipment	
		Compatibility	Inability to conduct group-based sessions		Inability to conduct group-based sessions and technological issues	
**TDF**						
	**Motivation**					
		Emotion	Patient discomfort or fear of in-hospital services and provider burnout		N/A	
		Beliefs about consequences	N/A		Patient safety	
		Beliefs about capabilities	N/A		Ability to remotely monitor home-based sessions or use telehealth devices	
	**Capability**					
		Knowledge	N/A		Unfamiliarity with home-based or telemedicine model	
		Decision-making	N/A		Triaging patients	

^a^N/A: not applicable.

#### Local Contextual Factors

Two key clinician stakeholders (both female; CR supervisor [PT]: n=1, 50%, and associate professor of rehabilitation and regenerative medicine [doctor of medicine], n=1, 50%) agreed to complete a series of in-depth interviews to enable us to understand the local context of CR and telehealth at Columbia University Irving Medical Center or NYPH. The barriers and facilitators to clinic- and home-based CR from step 1 were confirmed, with hybrid CR emerging as the ideal model to implement at NYPH (eg, the CR supervisor would “envision a hybrid type of program”). When asked about telehealth and documenting remote visits, both stakeholders mentioned that all video visits and data collection happen in the Epic cloud-based EHR (eg, “Everything is in Epic,” and “It’s all in the EMR [electronic medical record]”) and that the staff are familiar with technology, but learning to adopt a new system may be challenging (eg, “The good thing is that [the staff] is comfortable using the technology...logging on to MyChart isn’t unfamiliar, trying out a new way to do something is a barrier”). Contextual inquiry also revealed that the primary CR patient population in the Washington Heights neighborhood of New York City consists of predominantly Hispanic Spanish-speaking patients with varying levels of socioeconomic status and digital health literacy.

### Step 2 Results: Design of the Initial THCR Prototype

Informed by our design concept, 20 design intervention prototype workshops or meetings were conducted to design an initial nontraditional CR prototype that addressed the contextual (informed by the CFIR) and behavioral (informed by the TDF) determinants of CR implementation that emerged in step 1, as well as offered the same core components of traditional CR. Of these 20 sessions, 2 (10%) focused solely on nontraditional CR programming (eg, frequency of visits, exercise modality or equipment, and education content or materials), 7 (35%) focused solely on telehealth (eg, devices, platform, and remote monitoring), 6 (30%) focused on both nontraditional CR programming and telehealth, and 5 (25%) focused on eliciting feedback from additional stakeholders (eg, hospital leadership or administration and NYPH telehealth working group). A total of 7 follow-up email threads among design team members and additional stakeholders were used to facilitate prototype design between the workshops or meetings.

The co-design process revealed that the initial nontraditional CR prototype should include the following key design elements: (1) combination of home- and clinic-based CR sessions (ie, hybrid vs clinic based only), (2) fewer total number of CR sessions (ie, 24 vs 36 sessions), (3) reduction in total duration of direct clinician supervision or monitoring (eg, 20 minutes vs 60 minutes of provider supervision via 2-way video call during home-based sessions), (4) real-time remote patient monitoring (RPM) of resting and exercise vital signs (ie, synchronous vs asynchronous monitoring), (5) comparable home- and clinic-based exercise dose (frequency, intensity, and time), (6) alignment with existing clinical workflow and telehealth infrastructure (vs external processes or vendors), and (7) provision of training for clinicians and patients. Table S3 in Multimedia Appendix 1 outlines how each design element addressed the step 1 determinants (eg, reimbursement and compatibility).

Throughout the design process, details of the initial prototype design evolved based on stakeholder feedback, NYPH infrastructure, and ability of the proposed design elements to address user and contextual determinants; for instance, the commencing version of the initial prototype leveraged Epic Systems’ MyChart Video Visits, which aligned with existing clinical workflow and infrastructure but required patients to (1) have their own electronic device, (2) have access to Wi-Fi, and (3) independently log in to their Epic portal. Specifically, MyChart Video Visits did not support interoperability between commercial patient monitoring devices (eg, Fitbit, Polar HR monitor, and store-bought BP monitor) and the EHR, hindering the ability to provide real-time RPM during home-based sessions. Accordingly, we leveraged the expertise from our digital health team members and decided to partner with NYPH’s investment in Philips Healthcare’s RPM platform, which included (1) freely available RPM devices (eg, tablet device, pulse oximeter, and BP monitor and cuff) that wirelessly transmit BP and HR data to a web-based tracking database (eCareCoordinator [eCC]; Philips Healthcare) during CR sessions or exercise, (2) real-time integration of HR and BP data into the EHR (eCC interfaces with Epic), and (3) telemonitoring-enabled CR support via 2-way video calls. Moreover, to address key determinants highlighted by stakeholders in step 1 (eg, reimbursement and available resources), this platform was chosen to ensure that patients had access to reimbursable resources (vs out-of-pocket expenses) that support both cellular and Wi-Fi connectivity (vs requiring Wi-Fi access or a cellular data plan).

Ultimately, this process yielded an initial THCR prototype to offer 24 CR sessions, each lasting 60 minutes (with partial clinician supervision), over 12 weeks. The prototype combined home-based CR (eg, remote exercise monitoring) with NYPH’s existing clinic-based CR (ie, standard of care), EHR (ie, Epic), and telemonitoring (eg, Philips Healthcare RPM devices and eCC) infrastructure. Home-based exercise equipment included a stationary cycle-ergometer for aerobic exercise and ankle and wrist weights for strength training. Education and counseling about exercise, nutrition, psychosocial support, and risk factor management aligned with NYPH’s existing clinic-based CR programming, which included one-on-one counseling with CR clinicians during sessions and self-administered educational videos between sessions. The home-based CR protocol and telehealth monitoring platform from this initial prototype were user tested in step 3.

### Step 3 Results: Initial Prototype Refinement Based on Patient- and Provider-Level Usability

We conducted 8 usability sessions (multiple sessions per patient) to simultaneously troubleshoot the patient (n=3; female: n=1, 33%; Black: n=1, 33%; and Hispanic: n=2, 67%) and clinician (both female) experiences when using the eCC platform, RPM devices, and home-based exercise equipment, as well as patient-clinician communication during the sessions. As the end users engaged in >1 session throughout the program (ie, 12-week program with 24 sessions in total), multiple usability sessions were conducted per patient (patient 1 sessions: 4/8, 50%; patient 2 sessions: 2/8, 25%; and patient 3 sessions: 2/8, 25%). At least 1 clinician participated in each usability testing session. All sessions were conducted in English and aligned with real-world clinical workflow (eg, 60 minutes per session and scheduling). The NYPH design team completed intermittent design team workshops (n=24) and journey mapping sessions (n=3) to refine elements of the protocol and create a final prototype.

Thematic analysis of the usability testing observations and meeting minutes revealed patient- and clinician-level themes (*codes*) for different prototype intervention components (Table 2). Patient-level themes for RPM devices were related to the ease of using the devices (*capability/comfort using the devices, visibility,* and *navigation*) and technology disruptions (*Wi-Fi/​Bluetooth connectivity*)*,* whereas themes for exercise were related to comfort with ability to perform or use exercise modality or equipment (*capability/comfort* and *safety*) and flexibility with exercise experience (*adaptations/flexibility*). CR clinician–level themes for the eCC platform were ease of using the telehealth platform to remotely monitor patients (*visibility* and *navigation*), technology disruptions (*Wi-Fi/​Bluetooth connectivity*)*,* and confidence in using the telehealth platform to safely monitor patients (*confidence in technology* and *safety concerns*). Each of these themes aligned with key usability constructs and principles (eg, learnability, efficiency, and satisfaction; Table S4 in Multimedia Appendix 1). Accordingly, design solutions were identified and incorporated into the final prototype to improve the patient-facing experience with RPM and exercise (eg, onboarding support, safety protocol, and flexibility in programming based on patient progression) and clinician-facing experience with the eCC platform (eg, revise eCC feature layout and interface, provide training aids and technical support, and add safety check features to eCC protocol). Examples of the design solutions in relation to usability themes are outlined in Table S5 in Multimedia Appendix 1. As for general programming, we detected positive comments and feedback on the patient experience when using the devices and the process or workflow of the prototype. Details of the final prototype and changes made to the initial prototype based on step 3 are outlined in Table S6 in Multimedia Appendix 1. The refined or final THCR prototype that emerged from step 3 has been implemented at an NYPH CR clinic in the form of an NIH-funded pilot randomized controlled trial (RCT; UL1TR001873/​KL2TR001874); implementation and usability outcomes of the entire 12-week program design will be rigorously assessed using a mixed methods approach. Details of the pilot RCT have been registered on ClinicalTrials.gov (NCT05328375).

**Table 2:** Themes, codes, and representative examples of patient and clinician experiences during prototype usability testing (step 3).

Stakeholder, themes, and codes			Representative quotes and minutes from observations and meetings
**Patient**			
	**Ease of using the RPM^a^ devices**		
		Capability and comfort	“Using the pulse oximeter while on the bike was hard.”[patient 3]
		Visibility	Patient struggled to see the screen (of the tablet device) while on the bicycle. [patient 2]
		Navigation	Patient did not know that pressing the Philips icon opened the tablet device and did not know the PIN^b^. [patient 1]
	**Technology disruptions**		
		Wi-Fi, cellular, or Bluetooth connectivity	The (pulse oximeter measurements) were not syncing. At this point (the patient) verbally reported (their heart rate). [patient 1] The (pulse oximeter measurements) were not populating in real time. (The patient) removed (the pulse oximeter) and then replaced it on their finger. [patient 1] When using the tablet device for a 2-way video call, the patient said, “[The clinician] froze.” [patient 2]
	**Comfort with ability to perform and use exercise modality and equipment**		
		Capability and comfort	(The clinician) spent 3 to 4 minutes of (the session) helping the patient adjust (the bicycle). [patient 2] “[The bicycle] feels different than the treadmill.” [patient 1]
		Safety	The clinician stated, “[The patient] wants to make sure [the clinicians] could see them [while exercising on the bicycle].” [patient 1] The clinician stated, “[The patient] needs to make sure [they have] something sturdy to hold on to [during strength training].” [patient 1]
	**Flexibility with exercise experience**		
		Adaptations and flexibility	The clinician allowed a patient to try interval training on the bicycle. When asked, “What did you like the most?” the patient responded, “Interval training.” [patient 3] The clinician stated, “[G]ive flexibility based on patient needs.” [patient 1] The clinician discussed (with the patient) the importance of tailoring exercise to current energy levels. [patient 1]
**Clinician**			
	**Ease of using the telehealth platform to remotely *monitor* patient**		
		Visibility	During the eCareCoordinator Video Visit (Philips Healthcare): “It’s easy to view the patient with the eCC [eCareCoordinator; Philips Healthcare] video call platform.” [clinician 1] When starting the eCareCoordinator Video Visit: “There’s no way to tell [in the eCareCoordinator platform] that someone is waiting for you on the video call.” [clinician 2]
		Navigation	When monitoring the patient: the clinician could not toggle between the “video visit” tab and “trends” tab in the eCareCoordinator platform. [clinician 2]
	**Technology disruptions**		
		Wi-Fi, cellular, or Bluetooth connectivity	[S]yncing was an issue between pulse oximeter, cuff, and eCC [eCareCoordinator] platform...[clinician] had to enter information manually. [clinician 1] “[T]here seemed to be a longer delay between audio and video...there may be an issue with Wi-Fi strength.” [clinician 1] “[The pulse oximeter reading] seems slower today...want to [verbally] read me the numbers?” [clinician 2] The clinician had to enter the blood pressure measurement into the eCareCoordinator manually. [clinician 2]
	**Confidence in using the telehealth platform to safely *monitor* patient**		
		Confidence in technology	“The [blood pressure and pulse oximeter] readings [the clinicians] got were pretty accurate.” [clinician 1] “The BP [blood pressure monitor] seemed to work more accurately.” [clinician 2] “The pulse oximeter reading was fine.” [clinician 2]
		Safety concerns	Clinician instructed the patient that “more weight isn’t always better...we want to prevent injury.” [clinician 2] After the survey was completed, [the clinician] was waiting for the blood pressure measurement to come in [to the eCareCoordinator platform], but it never did. The clinician said, “I have no idea what he’s doing.” [clinician 2] [Clinicians] cannot see [the patient’s] feet to determine whether they are wearing appropriate footwear. [clinicians 1 and 2]
**Patient and clinician**			
	**Satisfaction**		
		Positive comments and feedback	The clinicians said that the patient “loved [the home-based model] and was ecstatic [with their experience].” [patient 1] The clinicians said that the patients “seem to like it...they like the equipment.” [patient 2] At the end of the user testing sessions, the patient said, “Thank you again for everything and for the devices.” [patient 1]
	**Programming**		
		Process or workflow	“[The patient] thinks the model works.” [patient 3] “The process itself [the patient] really loved.” [patient 1] The clinician felt that “[the session] was a bit of a learning curve with the patient—but they were happy to go through it.” [patient 2]

^a^RPM: remote patient monitoring.

^b^PIN: personal ID number.

## Discussion

### Principal Findings

This study provides an outline of how to apply UCD and IS principles and methods at the early stages of intervention development to design a nontraditional CR program in a real-world hospital setting. We used an iterative 3-step process to (1) identify user and contextual factors of CR implementation; (2) engage key stakeholders within a health care system to co-design a prototype that targets relevant implementation determinants while retaining core components of the evidence-based practice; and (3) address real-world usability concerns based on clinician and patient experiences. The CFIR and TDF helped to focus the identification of contextual- (outer setting and inner setting) and user-level (motivation and capability) implementation determinants, respectively, for general clinic- and home-based CR (step 1). Design team workshops and journey mapping enhanced our ability to design the initial prototype (step 2) and refine the nontraditional CR model based on usability themes that emerged from each patient- and clinician-facing experience during usability testing (step 3). Collectively, this iterative multilevel stakeholder–focused process yielded a theory-informed 12-week THCR program prototype. The final program prototype has been implemented at NYPH, where we are examining the program’s potential to optimize end-user experiences (eg, usability and satisfaction) and implementation potential (eg, feasibility, acceptability, and appropriateness) in a real-world hospital setting serving racially, ethnically, and socioeconomically diverse populations.

This is the first study to apply 2 complementary IS frameworks (ie, the CFIR and the TDF) to examine stakeholder perspectives on behavioral and contextual determinants of both home- and clinic-based CR implementation, an essential first step to help inform the iterative co-design process. Although ample pre–COVID-19 pandemic research exists on clinic-based CR determinants (eg, referral, transportation, and work or family schedule) [57], our theory-informed approach unveiled new pandemic-related barriers and facilitated simultaneous comparison of home- versus clinic-based CR implementation determinants. Although novel clinic-based CR barriers primarily included contextual factors linked to the inner (eg, inability to conduct group-based sessions) and outer (eg, social distancing guidelines) settings, as well as stakeholder emotion (eg, patient fear and provider burnout), key home-based CR barriers largely encompassed behavioral factors linked to motivation (eg, beliefs about capabilities) and capability (eg, decision-making), as well as ongoing reimbursement constraints. Despite these barriers, the pandemic presented unique contextual facilitators for home-based CR implementation (eg, hospital-wide telehealth initiatives and leadership buy-in). The distinct determinants that emerged at different levels for clinic- and home-based CR highlight the value of simultaneously evaluating behavioral and contextual factors using theory-informed IS frameworks because both are important to inform key design elements. Other studies have applied the CFIR and other theoretical frameworks to understand the determinants of nontraditional CR programs [58], but none have used these frameworks in program development as we have done in this study.

Engaging multilevel stakeholders in the design process (eg, design team workshops and usability testing) ensured that the final prototype embodied design elements responsive to patient-, provider-, and system-level perspectives and experiences; for instance, the reimbursement of home-based CR has been documented as a persistent structural barrier in the literature [13] and was highlighted by provider- and system-level stakeholders as a key barrier to address in this work. In October 2020, the Centers for Medicare & Medicaid Services added CR to the list of approved telehealth services, partially addressing key reimbursement considerations [13]. Nonetheless, this addition is temporary, and coding limitations exist, highlighting the need to develop strategies to overcome reimbursement limitations, such as aligning with telehealth services (eg, synchronous audiovisual monitoring) that support reimbursable billing codes [32,59]. This has been made increasingly possible by new hospital-wide telehealth initiatives and improved telehealth infrastructures. Accordingly, the use of a hybrid model that leveraged the hospital system’s telehealth infrastructure to offer real-time RPM (eg, telehealth platform and devices) emerged as an optimal nontraditional design element from both system- and provider-level stakeholders because it addressed select barriers to both clinic- and home-based CR models. By engaging patients and providers in usability testing, we were able to unveil that design solutions were needed (eg, onboarding support) in the refined prototype to improve the patient- and clinician-facing experiences with using the RPM devices and platform; these are design elements that may not have emerged had we only engaged 1 stakeholder type (eg, patient vs provider or system) in the design process.

A unique contribution of this study is the combination of theory-informed IS frameworks and usability testing methods to examine patient- and clinician-level experiences with an innovative *nontraditional CR model*. Although scientific calls have been made to use novel approaches to address the *research-practice gap* in CR [13], and comprehensive literature outlining best practices to combine and apply IS and UCD exists [25,26], few studies have applied these concepts to the design and implementation of nontraditional CR models. Other studies have applied user- and human-centered design and theories to guide the development of nontraditional CR programs and apps as well as intervention elements (eg, patient portals), but few have used these methods simultaneously, and none have applied IS principles to guide their design process; for instance, Joensson et al [60] performed a similar 3-step process that engaged multiple stakeholders to develop a theory-informed (self-determination theory) cardiac telerehabilitation web portal called the HeartPortal, which provided design features to support patient-clinician communication and was found easy to navigate by patients with heart failure. Similarly, Duff et al [61] engaged in a 2-phase process to create a theory-informed exercise rehabilitation mobile app for adults with cardiovascular disease. In phase 1, the authors conducted a systematic review to identify behavior change techniques, which informed the design of their app, followed by phase 2, wherein they conducted focus group user testing and feasibility testing [61]. Two other studies applied theories (eg, health belief model and theory of planned behavior) and used iterative usability testing to develop mobile apps designed to either facilitate the delivery of home-based CR [62] or self-management in secondary prevention [63]. Although each of these studies combined UCD and theory, their process solely engaged the patient stakeholder during product (eg, patient portal and mobile health app) usability testing and design evaluation as opposed to eliciting feedback from other end users, such as health care professionals, and did not assess contextual factors using theory-informed frameworks that could influence implementation. A lack of nonpatient stakeholder (eg, CR practitioners and health care organizations) perspectives has been documented as a pressing gap in the literature as it relates to nontraditional CR implementation [64] because these stakeholders play a vital role in real-world implementation.

By contrast, Funahashi et al [65] applied a rigorous multilevel UCD approach that aligned with hospital system needs to develop a technology-enabled, evidence-based remote CR program; however, the authors did not infuse theory into the design of their program. To enhance rigor and replicability, behavior change theories and frameworks should guide the design and development of future complex CR interventions, as well as their implementation strategies. Interestingly, none of the aforementioned studies discussed or addressed reimbursement, and most of the studies that used UCD methods were conducted outside the United States (eg, Ireland [61], Finland [66], Australia [67], and Denmark [60]), which may be a reflection of the different reimbursement policies and health care systems in which the programs were designed (eg, private nonprofit [Kaiser Permanente] vs public health care systems and European vs US health care systems). As demonstrated in our work and existing literature, reimbursement of nontraditional CR programs is an important contextual factor to address in the design process. Moreover, few of these studies addressed racial and ethnic and socioeconomically diverse populations.

### Strengths and Limitations

This study has several strengths. First, this study expands the literature on the use of theory-informed implementation frameworks to characterize multilevel determinants of CR implementation to inform intervention development. Moreover, we provide an applied example of using the CFIR and TDF as complements to each other in the context of CR. The use of these frameworks provides a foundation to develop future multilevel implementation strategies. Second, this is among the first studies to combine both UCD and IS methods at the early stages of nontraditional CR development [23]. Third and last, the entire design process aligned with the clinical workflow and telehealth infrastructure of the hospital system. However, these findings should be interpreted in the context of several limitations. First, patient stakeholders and relevant family members were not included as interviewees in step 1 or as members of the NYPH design team, which may have influenced our understanding of the CR implementation determinants that informed the initial prototype design as well as the iterative feedback that led to the refined prototype. To address this limitation, real-world CR patient stakeholders with diverse racial and ethnic and socioeconomic backgrounds were included in the usability testing sessions (step 3), and CR clinicians with >10 to 30 years of direct experience administering CR to our target patient population were included in the design team. Second, given the iterative QI nature of this study, the measurement of usability and implementation outcomes lacked formal assessment (eg, Acceptability of Intervention Measure [68]), hindering our ability to quantify whether the refined prototype improved these outcomes. Third, the semistructured interviews were not audio recorded, limiting our ability to produce verbatim transcripts. Fourth, usability testing occurred only for the initial (vs final) prototype among patients who spoke English (vs multiple languages) and did not reflect all program design elements (eg, number of sessions, combination of clinic- and home-based sessions, and education or nonexercise components), which may have limited the depth and inclusivity of feedback detected. To address these methodological concerns, rigorous quantitative and qualitative data on implementation determinants, implementation outcomes, and usability outcomes of the final prototype design are being collected and analyzed among English- and Spanish-speaking participants in our ongoing pilot RCT. Fifth and last, this is a small single-center cross-sectional study based on a QI project at an urban academic medical center, which may limit the generalizability of our findings. These limitations notwithstanding, our findings shed light on how an iterative multilevel-stakeholder combined IS and UCD process can inform the design of nontraditional CR programs in the telemedicine era.

### Future Directions

Additional research is needed to understand the optimal design by which a nontraditional CR model can improve multilevel CR implementation, patient and provider experiences, and clinical outcomes in racial and ethnic and socioeconomically diverse settings, with the goal to mitigate inequities that exist in access to CR. Accordingly, future research should consider equity recommendations (eg, focus on reach from the very beginning and use an equity lens for implementation outcomes) at the early stages of intervention development, as well as consider costs to implement and sustain the program in real-world settings [54]. Moreover, given the persistent dismal uptake of CR, future research should complement the traditional translational research pipeline with IS methods and frameworks integrated into the early stages of nontraditional CR intervention development [22,23], as well as evaluate the effectiveness of implementing nontraditional CR interventions at scale in routine clinical practice [64]. Our study provides an applied example of the former, and the results of our mixed methods pilot study will inform the feasibility, acceptability, appropriateness, usability, and satisfaction of THCR, as well as reassess multilevel determinants of implementation in the post–COVID-19 era, in a low socioeconomic status setting serving primarily racial and ethnic minority groups with Medicaid. A prospective hybrid type I effectiveness-​implementation RCT will evaluate the program’s implementation potential and effectiveness compared with traditional CR. To inform the best practice translation of effective nontraditional CR interventions into routine clinical practice, pragmatic implementation studies that target all key implementation constructs across all relevant stakeholder levels are needed [64].

### Conclusions

This paper provides an applied example for integrating UCD and IS principles into the early-stage development of a THCR model while aligning with the behavioral and contextual factors of a real-world clinical setting. We found that IS frameworks can help to identify user and contextual factors of CR implementation; design team workshops and journey mapping can engage key stakeholders within a health care system to co-design a prototype that targets relevant implementation determinants while retaining core components of the evidence-based practice; and patient- and clinician-level usability testing can unveil real-world usability concerns to be addressed when refining the prototype. This process may serve as a useful model for future CR clinics that aim to design and implement a nontraditional CR program for their specific organizational or health care infrastructure and patient population.

## Appendix

Multimedia Appendix 1 User-centered design (UCD) and implementation science (IS) methods, stakeholder characteristics, nontraditional cardiac rehabilitation (CR) design elements, user-testing themes, design solutions and revisions, and journey map.

## Abbreviations

BP: blood pressure
CFIR: Consolidated Framework for Implementation Research
CR: cardiac rehabilitation
eCC: eCareCoordinator
EHR: electronic health record
HR: heart rate
IS: implementation science
NIH: National Institutes of Health
NYPH: NewYork-Presbyterian Hospital
PT: physical therapist
RCT: randomized controlled trial
RPM: remote patient monitoring
SQUIRE 2.0: revised Standards for Quality Improvement Reporting Excellence
TDF: Theoretical Domains Framework
THCR: telehealth-enhanced hybrid cardiac rehabilitation
UCD: user-centered design

## Data Availability

The data sets generated and analyzed during this study are not publicly available to protect stakeholder anonymity but are available from the corresponding author on reasonable request.
