# Supplementary material for: Applying User-Centered Design and Implementation Science to the Early-Stage Development of a Telehealth-Enhanced Hybrid Cardiac Rehabilitation Program: Quality Improvement Study
Source: JMIR Form Res. 2023 Jul 13;7:e47264. doi: 10.2196/47264 (PMC10375395; doi:10.2196/47264)

**Supplemental Materials**

**Title:** Applying user-centered design and implementation science to the early-stage development of a telehealth-enhanced hybrid cardiac rehabilitation program: quality improvement study

**Authors:** Andrea T. Duran^1^, PhD, MPhil, MS, Adrianna Keener-DeNoia^1^, MA, Kimberly Stavrolakes^2^, PT, MS, Adina Fraser^2^, MHA, Luis V. Blanco^1^, BA, Emily Fleisch^2^, PT, DPT, Nicole Pieszchata^2^, PT, DPT, Diane Cannone^1^, MPH, Charles Keys McKay^1^, BA, Emma Whittman^3^, BA, MS, Donald Edmondson^1^, PhD, MPH, Rachel C. Shelton^3^, ScD, MPH, Nathalie Moise^1^, MD, MS

^1^Columbia University Irving Medical Center, New York, NY

^2^New York Presbyterian Hospital, New York, NY

^3^Mailman School of Public Health, New York, NY

**Corresponding Author:**

Andrea T. Duran

Columbia University Medical Center

622 West 168^th^ Street

New York, NY 10032

Phone: 212-678-3325

Email: atd2127@cumc.columbia.edu

**Table S1. Overview of user-centered design (UCD) and implementation science (ImS) methods**

|  |  |  |
| --- | --- | --- |
| **Item** | **Description** | **Field** |
| ***Methods*** |  |  |
| Semi-structured Interviews | Semi-structured interviews include interview guides with specific questions that can be asked in a conversational (vs. formal) manner and do not need to be asked in sequential order as outlined in the guide. To accommodate the rapid and iterative nature of implementation research, semi-structured interview guides focus on questions that participants will likely be able to answer due to their expertise and role as it relates to the topic of interest/exploration [45]. | ImS & UCD |
| Consolidated Framework for Implementation Research (CFIR) | The CFIR is an overarching theoretical framework that specifies 39 standardized implementation-related constructs within five general domains (intervention, inner setting, outer setting, individuals, process of implementation) that can be applied across different implementation research phases and organizational levels [38]. | ImS |
| Theoretical Domains Framework (TDF) | The TDF is a comprehensive, theory-informed approach that consists of 84 theoretical constructs within 14 domains (e.g., knowledge, skills, social/professional role) that can be used to identify determinants of individuals’ behavior (e.g. provider, patient), enabling researchers to assess implementation problems and support intervention design [35]. | ImS |
| Contextual Inquiry | Contextual inquiry includes in-depth data on a few carefully selected individuals to inform a fuller understanding of users and their context [41]. | UCD |
| Design Team Prototyping Workshops | Design Team Prototyping Workshops engage a multidisciplinary group of prospective users and other stakeholders to generate design solutions based on project data (e.g., usability testing). This method complements implementation research by providing opportunities for the same group of users to review prototypes at multiple time points to collaboratively generate limited versions of the intervention/product [42]. | ImS & UCD |
| Journey Mapping | Journey mapping is a method that supplements the prototyping process by enabling visualization of the end-user (e.g., patient, provider) experience as they move through various interactions or touch points from beginning to end of their overall experience [43,44]. | UCD |
| Usability Testing | Usability testing involves evaluation of potential real-world end-users (e.g., patients, providers) interacting with/using products or services and completing representative tasks [46]. | UCD |
| Observations | Observations can be conducted for a variety of reasons, such as assessing local contexts, understanding stakeholder experiences with interventions, and the nature and intensity of how interventions are being implemented [48]. They often occur during site visits, wherein an evaluation team typically documents data in the form of field notes [49]. | ImS |
| Archival Analysis | Archival analysis involves the compilation of documents, including meeting minutes, guidelines, local data, policies, and health records [47,50]. | ImS |
|  |  |  |

**Table S2.** Stakeholders involved in the design process**.**

| **Professional Title** | **Degree** | **Gender** | **Stakeholder Type** | **Semi-structured interview** | **Contextual Inquiry** | **Design Team Members** | **Usability Testing** |
| --- | --- | --- | --- | --- | --- | --- | --- |
| Patient Navigator | Not Reported | Female | CR Staff | X |  |  |  |
| Clinical Coordinator Cardiac Rehab | MS | Male | CR Clinician | X |  |  |  |
| Registered Nurse | MSN, RN, A-CFHC | Female | CR Clinician | X |  |  |  |
| Director, Preventive Cardiology Unit | PhD | Male | CR Director | X |  |  |  |
| Director, Rehabilitation Medicine | MD | Male | Health System Leader | X |  |  |  |
| Site Director, Physical Therapy | PT, MA | Female | Health System Leader | X |  |  |  |
| Supervisor, Cardiopulmonary PT | PT, MS | Female | CR Supervisor | X | X | X | X |
| Senior Physical Therapist | PT, DPT | Female | CR Clinician | X |  | X | X |
| Advanced Clinician, Outpatient Cardiopulmonary Rehab | PT, DPT | Female | CR Clinician | X |  | X |  |
| Associate Professor of Rehabilitation and Regenerative Medicine | MD | Female | Provider with expertise in telehealth and rehabilitation medicine |  | X |  |  |
| Project Lead, Digital Health | MHA | Female | Digital Health |  |  | X |  |
| Sr. Project Coordinator, Digital Health | B.Tech | Female | Digital Health |  |  | X |  |
| Creative Director | BA | Male | Design |  |  | X |  |
| Teacher | Not Reported | Male | CR Patient |  |  |  | X |
| Hospital Staff | Not Reported | Female | CR Patient |  |  |  | X |
| Attorney | Not Reported | Male | CR Patient |  |  |  | X |

**Table S3.** Nontraditional cardiac rehabilitation (CR) design elements mapped onto multi-level determinants of implementation.

|  |  | **Nontraditional CR Design Elements** | | | | | | |
| --- | --- | --- | --- | --- | --- | --- | --- | --- |
| **Determinants** | **Home-based CR** | **Clinic-based CR** | **Telehealth/Philips RPM program** | **Comparable Exercise** | **Reduce # and duration of sessions** | **Training** | **Leverage existing infrastructure** | **Collaborate with champions/leaders** |
| Social distancing guidelines | X |  | X |  |  |  |  |  |
| Reimbursement |  | X | X |  |  |  |  |  |
| Overwhelmed healthcare system |  |  |  |  | X |  |  |  |
| Non-essential service |  |  |  |  |  |  | X | X |
| Provider redeployment |  |  |  |  |  |  | X | X |
| Limited number of staff |  | X |  |  | X |  |  |  |
| Limited physical space | X |  |  |  |  |  |  |  |
| Limited staff capacity |  |  |  |  | X |  | X |  |
| Telehealth services/devices |  |  | X |  |  |  |  |  |
| Home-based exercise equipment |  |  |  | X |  |  |  | X |
| Wi-Fi access |  |  | X |  |  |  |  |  |
| Inability to conduct group-based sessions |  |  |  |  | X |  |  |  |
| Patient discomfort/fear of in-hospital services | X |  |  |  |  |  |  |  |
| Provider burnout |  | X |  |  | X |  | X |  |
| Patient safety |  | X | X | X |  |  | X |  |
| Ability to remotely monitor home-based sessions/use telehealth devices |  |  |  |  |  | X |  |  |
| Unfamiliarity with home-based CR/telemedicine |  |  |  | X |  | X |  |  |
| Triaging patients |  |  |  |  |  | X |  |  |

**Table S4.** User-testing themes mapped onto usability constructs that reflect user-centered design principles.

|  |  | **Usability Constructs** | | | | | |
| --- | --- | --- | --- | --- | --- | --- | --- |
| **Intervention Component** | **User-Testing Theme** | **Learnability** | **Efficiency** | **Memorability** | **Error Reduction** | **Satisfaction** | **Exploit natural constraints** |
| RPM devices | Ease of using the RPM devices | X | X |  |  |  |  |
|  | Technology disruptions |  |  |  |  |  | X |
| Exercise | Comfort with ability to perform/use exercise modality and equipment | X |  |  | X |  |  |
|  | Flexibility with exercise experience |  |  |  |  | X |  |
| eCC platform | Ease of using the telehealth platform to remotely monitor patient |  | X |  |  |  |  |
|  | Technology disruptions |  |  |  |  |  | X |
|  | Confidence in using telehealth platform to safely monitor patient |  |  |  | X | X |  |
| General Programming | Satisfaction |  |  |  |  | X |  |
|  | Programming |  | X |  |  |  |  |
| Notes: eCC= eCareCoordinator, RPM= remote patient monitoring. | | | | | | | |

**Table S5.** Design solutions from Step 3 mapped onto user-testing themes.

| **Design Solutions (Stakeholders)** | **User-Testing Theme** | **Examples** |
| --- | --- | --- |
| Onboarding Support  (Patient) | Ease of using the RPM devices | Provide binder with written instructions on how to use RPM devices.  In-person onboarding session to (1) introduce RPM devices, (2) demonstrate how to use RPM devices, and (3) have patient practice using the RPM devices. |
|  | Technology disruptions | Remote onboarding session to confirm RPM devices and features (e.g., video call audio, measurements, surveys) are working from home-based environment. |
|  | Comfort with ability to perform/use exercise modality and equipment | Provide a binder with written instructions on how to set up and adjust the exercise equipment.  In-person onboarding session to (1) introduce exercise equipment and modality, (2) demonstrate how to use exercise equipment, and (3) have patient practice using the exercise equipment. |
| Training & Technical Support (Clinician) | Ease of using the telehealth platform to remotely monitor patient | Provide CR clinicians with formal Philips Healthcare training on how to use eCC platform to enroll and monitor patients.  Provide CR clinicians with visual aids to navigate the eCC platform. |
|  | Technology disruptions | Develop process to connect clinicians with Philips Healthcare representative to troubleshoot issues as needed. |
| Safety protocol and materials (Patient and Clinician) | Comfort with ability to perform/use exercise modality and equipment | Provide patients with fully supervised remote sessions at the beginning of the program to ensure safety.  Provide patients with an outline of the safety protocol. |
|  | Confidence in using telehealth platform to safely monitor patient | Administer pre- and post-exercise surveys to confirm patient location and well-being in the case of an emergency. |
| Flexibility in programming based on exercise progression (Patient) | Flexibility with exercise experience | The rating of perceived exertion target will vary week to week depending on the patient’s progression throughout the program.  The assigned weight (lbs) and number of repetitions per exercise will vary week to week depending on the patient’s progression throughout the program. |

**Table S6.** Revisions reflected in the final prototype based on feedback from Step 3 of the design process.

| **Design Elements (User)** | **Clinic-Based Sessions** | **Home-based Sessions** | **Revision** |
| --- | --- | --- | --- |
| Total Number of Program Sessions  (Patient & Clinician) | 5 | 19 | Additional clinic-based CR session and one less home-based CR session |
| Frequency of Sessions  (Patient & Clinician) | 2 sessions the first week  1 per month thereafter | 2/week | First week of the program includes clinic-based CR sessions (vs. fully remote) |
| Duration of Sessions  (Patient) | 60 minutes | 60 minutes | Feedback did not suggest change |
| Duration of Monitoring Sessions (Clinician) | 60 minutes | 60 minutes (first 2 sessions)  20 minutes (each subsequent session) | First week of home-based CR sessions are fully supervised (vs. partially supervised) |
| Patient Monitoring Platform,  Mode of Supervision  (Patient & Clinician) | Direct, In-person | Remote, Philips eCC/Samsung Video Visits | Feedback did not suggest change |
| Patient Monitoring Devices  (Patient) | Electrocardiogram machine, Blood Pressure Cuff and Monitor | Pulse Oximeter, Blood Pressure Cuff and Monitor, Samsung Tablet | Feedback did not suggest change |
| Aerobic Exercise  (Patient) | Modality: Walk/Jog  Equipment: Treadmill | Modality: Cycle  Equipment: Stationary bike | Feedback did not suggest change |
| Strength Training  (Patient) | Modality: Upper and Lower Body Exercises  Equipment: Dumbbells | Modality: Upper and Lower Body Exercises  Equipment: Ankle/Wrist weights | Feedback did not suggest change |
| Training/Support  (Clinician) | Standard of Care | Philips Training, Tech Support, and Cheat Sheets | Provide CR clinicians with adequate training, tech support, and visual aids |
| Training/Support  (Patient) | Standard of Care | 2 onboarding sessions (1 in-clinic and 1 remote) prior to starting home-based sessions  Onboarding binder w/ instructions | Provide patients with structured training and onboarding support outside of their scheduled CR sessions |
| Safety (Patient) | Standard of Care | Verbally confirm patient location  Surveys to confirm location & well-being | Additional safety checks at the beginning and end of each CR session |
| Notes: CR= cardiac rehabilitation, EHR= electronic health record, eCC= eCareCoordinator. Cells highlighted in blue indicate design elements of the initial prototype that were revised based on Step 3. | | | |

**Figure S1.** Journey map of the patient-level experience during a fully supervised home-based cardiac rehabilitation session


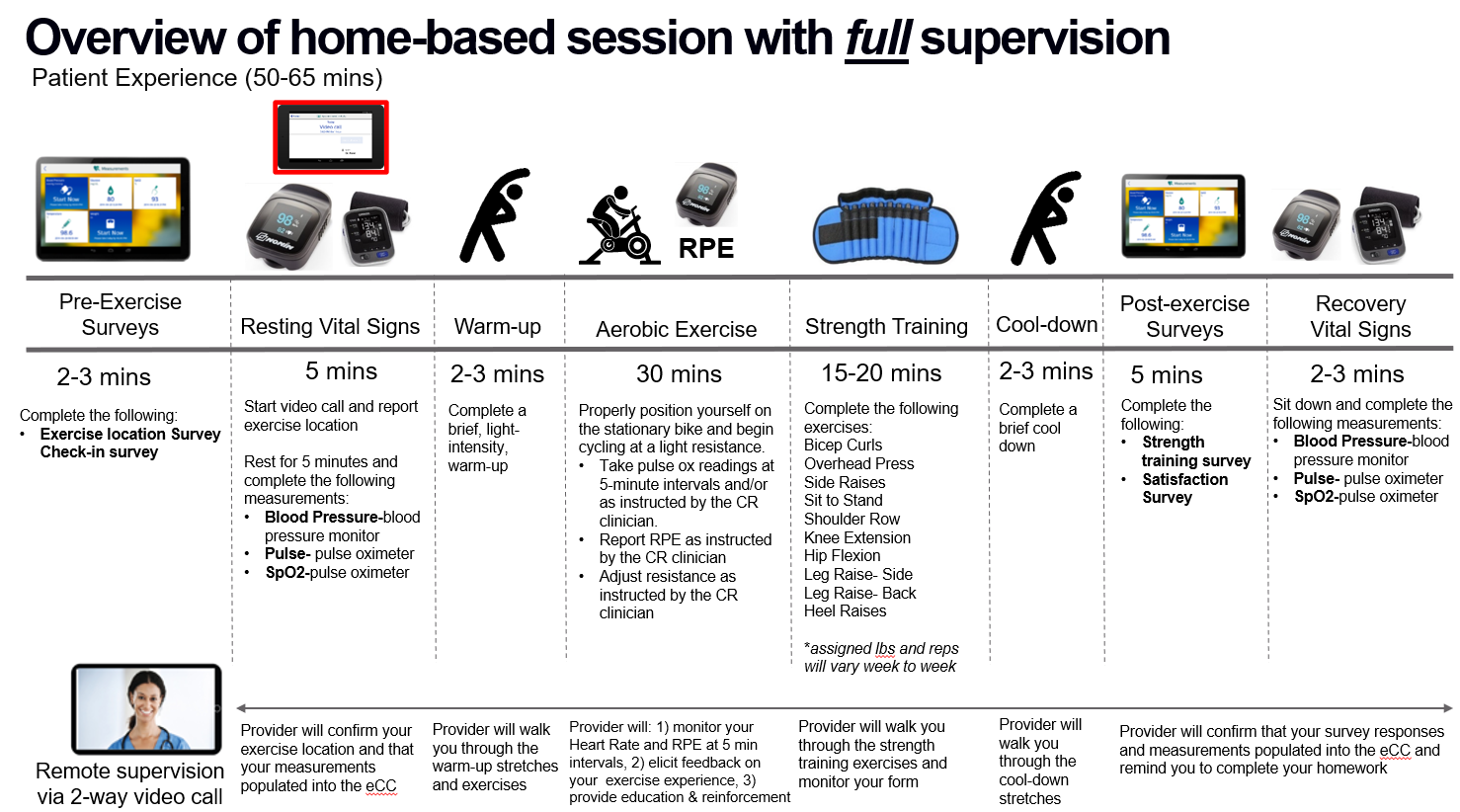

Supplement: Multimedia Appendix 1 [file formative_v7i1e47264_app1.docx]
